# Identification and Characterisation CRN Effectors in *Phytophthora capsici* Shows Modularity and Functional Diversity

**DOI:** 10.1371/journal.pone.0059517

**Published:** 2013-03-25

**Authors:** Remco Stam, Julietta Jupe, Andrew J. M. Howden, Jenny A. Morris, Petra C. Boevink, Pete E. Hedley, Edgar Huitema

**Affiliations:** 1 Division of Plant Sciences, University of Dundee, Invergowrie, Dundee, United Kingdom; 2 Dundee Effector Consortium, The James Hutton Institute, Invergowrie, Dundee, United Kingdom; 3 Cell and Molecular Sciences, The James Hutton Institute, Invergowrie, Dundee, United Kingdom; University of the West of England, United Kingdom

## Abstract

*Phytophthora* species secrete a large array of effectors during infection of their host plants. The *Crinkler* (CRN) gene family encodes a ubiquitous but understudied class of effectors with possible but as of yet unknown roles in infection. To appreciate CRN effector function in *Phytophthora*, we devised a simple *Crn* gene identification and annotation pipeline to improve effector prediction rates. We predicted 84 full-length CRN coding genes and assessed CRN effector domain diversity in sequenced Oomycete genomes. These analyses revealed evidence of CRN domain innovation in *Phytophthora* and expansion in the *Peronosporales*. We performed gene expression analyses to validate and define two classes of CRN effectors, each possibly contributing to infection at different stages. CRN localisation studies revealed that *P. capsici* CRN effector domains target the nucleus and accumulate in specific sub-nuclear compartments. Phenotypic analyses showed that few CRN domains induce necrosis when expressed *in planta* and that one cell death inducing effector, enhances *P. capsici* virulence on *Nicotiana benthamiana*. These results suggest that the CRN protein family form an important class of intracellular effectors that target the host nucleus during infection. These results combined with domain expansion in hemi-biotrophic and necrotrophic pathogens, suggests specific contributions to pathogen lifestyles. This work will bolster CRN identification efforts in other sequenced oomycete species and set the stage for future functional studies towards understanding CRN effector functions.

## Introduction

Plant pathogenic oomycetes continue to hamper crop production and damage ecosystems on a global scale. Perhaps the most notorious group of pathogens are found within the *Phytophthora* genus, where member species such as *Phytophthora infestans* and *Phytophthora sojae* wreak havoc on potato, tomato and soybean crops, whilst others such as *Phytophthora ramorum*, *Phytophthora kernoviae* and *Phytophthora lateralis* are rapidly emerging pathogens of trees, increasingly affecting forests and ecosystems. There is an urgent need to understand the mechanisms underpinning parasitism in this important group of eukaryotes, an undertaking that has sparked genome-sequencing efforts on a number of oomycete species [1]. With oomycete genome sequences available covering a broad spectrum of lineages and lifestyles, the challenge is to translate oomycete gene repertoires into the basic biology underpinning infection, virulence and pathogenic lifestyles.

*Phytophthora* spp are hemi-biotrophic pathogens that feature biotrophy early in infection and necrotrophy in the later stages of host tissue colonization. Both sporangia and the motile spores they produce (zoospores) can germinate and produce hyphae that penetrate the plant epidermis and invade host tissue. Pathogen ingress is followed by formation of specialized structures (haustoria) that invaginate living host cells (biotrophy) and support further pathogen growth and colonization of host tissues. Colonization ultimately leads to cell death and tissue collapse (necrotrophy) and in those later stages of disease development, sporangia are formed to initiate the next disease cycle [2].

Plant pathogens secrete arsenals of proteins (effectors) that enable parasitic infection and reproduction [3], [4], [5], [6]. Plants perceive Pathogen Associated Molecular Patterns (PAMPs) upon which Pattern Triggered Immunity (PTI) is mounted. To counter PTI, successful pathogens have evolved large and diverse effector repertoires that can suppress PTI and trigger susceptibility (Effector-Triggered Susceptibility, ETS) [7], [8].

In addition to extracellular effectors that counter defence associated molecules in the host apoplast, *Phytophthora* species secrete and translocate effectors, termed RXLRs, across the haustorial host-pathogen interface where they target resident host proteins and cellular processes to enhance susceptibility. Translocation requires the presence of a signal peptide, followed by a conserved N-terminal RXLR motif [9], [10], [11], features which allow rapid identification of effector candidates from oomycete genome sequences. Consequently, RXLR effector repertoires have been easily identified in sequenced oomycete species, allowing rapid insights into their virulence functions [6]. Genome sequence and functional analyses have revealed that besides the RXLR effector class, *Phytophthora* genomes encode another class of host-translocated effectors. The Crinkler (CRN for CRinkling and Necrosis) protein family was identified and named after a characteristic leaf crinkling phenotype observed upon ectopic expression of *P. infestans* secreted proteins in plants [12]. Critically, expressed mature CRN proteins retained cell death-inducing activity, suggesting functions targeting cytoplasmic host factors, a hypothesis that was confirmed when translocation activity of CRN N-termini, carrying an LXLFLAK motif, was demonstrated [13].

Unlike RxLR effectors, CRNs are present in all plant pathogenic oomycete species sequenced to date [13], [14], [15], [16], [17], [18], [19]; and this study). Over 196 Full length CRN-genes and 255 pseudogenes have been predicted from the *P. infestans* genome [16]. In other sequenced species, CRN predictions range from a total of 60 for *P. ramorum* to 202 for *P. sojae,* whereas much lower numbers (26) have been described in *Pythium ultimum* [17]. All share a conserved N-terminal domain with a characteristic LXLFLAK motif, however this domain is slightly altered in *Albugo candida* to LYLAK [18]. Interestingly, the LXLFLAK motif in some *Hyaloperonospora parasitica* CRN proteins are fused with RXLR motifs, suggesting they share ancestors [20]. In contrast to CRN N-termini, CRN C-terminal domains feature high levels of variation. Interrogation of the *P. infestans* genome sequence combined with analyses of other *Phytophthora* CRN effector complements, helped define and classify diverse C-terminal effector domains in *Phytophthora* species [16]. Interestingly, transient expression of CRN C-termini in plants, cause cell death in some cases, suggesting effector-mediated perturbation of host cellular processes. Indeed, subsequent studies have demonstrated a role for some CRN C-termini towards *P. sojae* virulence on soybean [21]. Although the exact functions have not been defined, recent studies demonstrated that at least one CRN effector domain in the *P. infestans* CRN8 C-terminus exhibits kinase activity, suggesting a role in modifying host signalling cascades during infection [22], [23].

Recently, the genome of the broad host range pathogen *Phytophthora capsici* was completed [19]. Automated gene identification and subsequent annotation revealed the presence of a relatively small number of CRN coding genes. This observation, together with a limited understanding of CRN effector distribution and function in *Phytophthora* and other oomycete species, prompted us to identify, validate and study the full *P. capsici* CRN effector domain repertoire in more detail. We applied a simple but robust pipeline to identify 84 full-length CRN protein candidates in *P. capsici* and used this validated set of gene models to assess the occurrence of CRN domains in other sequenced oomycete species. Our results suggest dramatic expansion of effector domains in hemi-biotrophic oomycetes, suggesting CRN effector innovation for hemi-biotrophy. Despite CRN effector domain conservation across *Phytophthora* clades, we defined species-specific effector domains and combinations, providing evidence for recent evolution in this protein family. Consistent with the idea of CRN involvement in pathogenesis, we confirmed expression of most CRN coding genes during infection and defined two sub-classes of effectors based on contrasting gene expression patterns. Localisation studies showed that all tested eGFP-CRN fusion-proteins accumulate in the nucleus and some exhibited specific subnuclear localisation patterns. These results suggest that targeting of host nuclear and subnuclear factors is an important requirement for infection. We substantiated these results with functional characterisations that indicate specific roles for CRN proteins towards *P. capsici* virulence. This work will bolster CRN identification efforts in other sequenced oomycete species and set the stage for future studies towards understanding CRN effector functions.

## Methods

### CRN Identification and Annotation

Databases: Phyca11 scaffolds, gene models and proteins, were obtained from the *Phytophthora capsici* sequencing consortium website ([19]; http://genome.jgi-psf.org/Phyca11/Phyca11.home.html). Databases for other oomycete species were obtained from their original sources: http://genome.jgi-psf.org for *P. ramorum* and *P. sojae*, genome.wustl.edu/for *Hyalopernospora. arabidopsidis,* http://pythium.plantbiology.msu.edu/for *Pythium ultimum,* http://www.polebio.scsv.ups-tlse.fr/aphano/for *Aphanomyces euteiches. Albugo candida* sequences were obtained from Matthew Links (Agriculture and Agri-Food Canada). *Pseudoperonospora cubensis* genomic data are described by Savory *et al* [24] and are, together with *Saprolegnia parasitica* data, available from NCBI. *P. infestans, P. sojae and P. ramorum* reference data and models were previously described [16]. *Phaeodactylum tricornutum* [25] and *Thalassiosira pseudonana* [26] genomes were obtained from JGI. *Arabidopsis thaliana* data was downloaded from TAIR, *Solanum lycopersicum* data from the SOL genomics consortium.

Annotations: ORFs were selected from Phyca11 scaffolds and basic sequence modifications were performed using EMBOSS (getorf –minsize 300) and bundled in a database (seqret). To identify CRN candidates we used BLAST (tblastp, –E 1e−4) [27] with 16 PiCRN aa sequences and HMMer3.0 (hmmsearch, –E 1e−4) on extracted ORFs.

HMMer was used to investigate domain presence C-terminal domains and for analysis of domain orientation. Manual alignment in jalview [28] was performed for curation of the final sequence and to see if identified CRNs matched our full length criteria (full length sequences should contain domains LFLAK(DI)DWL and a match with a C-terminal domain for at least 80% of the length of the shortest known variant in *P. infestans, P. ramorum* or *P. sojae*). We used the domain nomenclature as previously [16]. Sequences that did not match previously described domains were manually aligned and clustered to form new domains. Searches for specific functional domains and localisation domains were done using pFAM [29], NLStradamus [30], PredictNLS [31], Nod [22], SignalP3.0 [32], TMHMM [33] and the results were stored in our CRN library (Table S01) and were uploaded to the *P. capsici* genome database (http://genome.jgi-psf.org/Phyca11). To analyse domain evolution we searched protein models (or translated transcripts) from the oomycte databases mentioned above using both existing and new CRN HMM profiles (hmmsearch -E 1e−5).The Sequence logo was made using weblogo (http://weblogo.berkeley.edu/logo.cgi) and Venn diagrams with Venny (http://bioinfogp.cnb.csic.es/tools/venny/index.html).

### Microarray Analysis

Microarray data was generated from a *Phytophthora capsici*-tomato time course infection experiment (Jupe *et al.,* in prep). A custom Agilent 60-mer oligonucleotide microarray was designed from predicted *P. capsici* (LT1534 v11.0) and *Solanum lycopersicum* (ITAG 2.3) sequences using eArray software (https://earray.chem.agilent.com/earray/). The design is available at ArrayExpress (accession A-MEXP-2253; http://www.ebi.ac.uk/arrayexpress/) and represents 20,530 transcripts for *P. capsici* and 34,510 transcripts for *S. lycopersicum*. All RNA labeling and microarray hybridisation procedures were performed in the Genome Technology lab, James Hutton Institute, using standard operating procedures. Total RNA was extracted as described above, quantified by a NanoDrop ND-100 spectrophotometer (NanoDrop Technologies, USA) and quality checked using a RNA 6000 Nano Kit on a Bioanalyzer (Agilent Technologies). Fluorescent one-colour labeling of the RNA was performed as recommended (Agilent One-Color Microarray-Based Gene Expression Analysis (Low Input Quick Amp Labeling) v. 6.5) using 8×60 k format slides. Following array scanning, images were first imported into Agilent Feature Extraction (FE v. 10.7.3.1) software, aligned and quality checked using a corresponding grid template file. The microarray experimental design, along with raw datasets is available at ArrayExpress (accession E-MTAB-1295). The extracted FE dataset was separated for each array into *P. capsici* and *S. lycopersicum* data to allow independent processing. Datasets were each independently quality filtered using flag values (present or marginal in 2/3 replicates) and then quantile normalised in Genomics Suite software (Partek), prior to loading into Genespring (Agilent v. 7.3) software for analysis. The *Crn* gene set was extracted from the dataset and filtered based on replicated minimum raw expression values (>50) and normalised values (>1) in more than two stages, with at least a 2log change between stages. Genes were clustered using k-means (100 iterations) and gene trees were constructed using Pearson correlation and single linkage (default settings).

### PCR and Cloning of CRNs

For gene confirmation, PCR primers were designed for a selection of predicted full length CRN coding genes. PCR reactions were performed on cDNA derived from RNA that was extracted from infected *Nicotiana benthamiana* leaves using Gotaq polymerase (Promega). Amplicons were purified and Sanger sequenced on ABI3730 using Big Dye labelling chemistry.

For gene expression *in planta*: PCR primers were designed to specifically amplify selected individual CRN C-termini (Table S04). For CRNs 1_719, 11_767, 20_624, 79_188 and 83_152 the primers were designed to contain restriction sites compatible with pENTR1A (Life Technologies). For cloning, PCR was done using Phusion proofreading polymerase (New England Biolabs) on cDNA samples described above. The purified PCR products were ligated after appropriate restriction digestion using T4 DNA ligase. The entry plasmids were transformed using electrotransformation into *E. coli* DH10B cells.

Primers for the additional CRNs were designed to contain a CACC sequence to allow for GATEWAY directional TOPO cloning in pENTR-D-TOPO (Life Technologies). We cloned *Crn* genes following the manufacturer’s instructions and transformed the entry plasmid into *E. coli* MACH1 cells (Life Technologies). Primers were designed to add a small strep-II tag [34] to the C-terminus of each CRN protein. Colonies carrying the correctly sized inserts were grown overnight in liquid LB medium and inserts were sequenced (Table S04). ENTRY vectors were recombined into pB7WGF2 [35] using LR clonase II (Life Technologies) following the manufacturer’s protocol and transformed into electrocompetent *E.coli* DH10B. Colony PCR was done using M13-Forward and one CRN specific primer to verify the presence of each insert.

### Transient Expression of CRNs

Transient expression: pB7WGF2 plasmids containing CRN inserts, were transformed into *Agrobacterium tumefaciens* strain AGL1. Transformants were grown on LB medium containing Rifampicin and Spectinomycin to maintain each plasmid. For each construct, a single colony was grown overnight and resuspended in infiltration buffer (10 mM MgCl, 150 uM Acetosyringone) to an OD of 0.1 for confocal microscopy, 1.0 for necrosis and 0.5 for growth assays. The buffer was mixed 1∶1 with buffer containing *Agrobacterium* expression silencing suppressor P19 and infiltrated in *N. benthamiana* leaves. Plants were grown in a glasshouse under 16 hours light and set at 26°C by day and 22°C by night.

### Phenotypic Assays

For each CRN construct, three sites were infiltrated in different leaves and for each CRN the infiltration was repeated 3 times. The level of cell death was scored after 4 days using a 1–6 scale, with one indicating no symptoms and 6 signifying severe (black) necrotic lesions.

*P. capsici* growth assays were done on leaves that were fully infiltrated with *Agrobacterium* strains carrying CRN constructs. Two days after infiltration, leaves were drop inoculated with two 10 µL droplets of zoospore solution (500,000 spores per mL). Lesion diameters were measured 2 days post inoculation (DPI).

### Confocal Microscopy

All localisation studies were done on *N. benthamiana* leaves transiently expressing GFP-tagged CRN C-termini, two days after infiltration. To maintain cell structure after detachment, the leaves were infiltrated with water before mounting on a microscope slide. Subnuclear localisation was examined in stable transformants of *N. benthamiana* expressing RFP-Fibrillarin [36]. Leaf samples were imaged using a Leica SP2 confocal microscope. The excitation wavelengths used were 488 nm for GFP and 561 nm for RFP.

### Western Blots

Plant tissues were harvested at 3 dpi. Protein extractions were done using GTEN buffer (10% Glycerol, 25 mM Tris, 1 mM EDTA, 150 mM NaCl) supplemented with 2% PVPP, 10 mM DTT and 1X Complete protease inhibitor cocktail (Roche). Samples were pulled down using a GFP-trap (Chromotek) and run on 12% SDS PAGE gels before transfer to PVDF membranes. Blots were blocked for 30 minutes with 5% milk in TBS-T (0.1% tween), probed with StrepII-HRP antibody (Genscript) and washed 3 times in TBS-T for 5 minutes before incubation with Millipore Luminata Forte substrate or SuperSignal West Femto (Pierce). Images were collected on a Biorad Geldoc Imager.

## Results

### A Simple Gene Annotation Pipeline Improves CRN Effector Identification Rates in *Phytophthora capsici*

We aimed to classify the full *P. capsici* CRN complement from the recently published *P. capsici* genome sequence. BLAST based searches of the publicly available Phyca11 gene model set, helped identify twenty-nine full length and seventy CRN-like pseudogenes [19]. Given the high percentage of CRN pseudogenes in *P. capsici* (73%) compared to *P. infestans* (56%) or *P. sojae* (50%) and considering the relatively low abundance of CRN-like gene models compared to other *Phytophthora* sp. with similar genome size, we asked whether we could improve prediction rates for *P. capsici* CRN coding genes.

**Figure 1:**
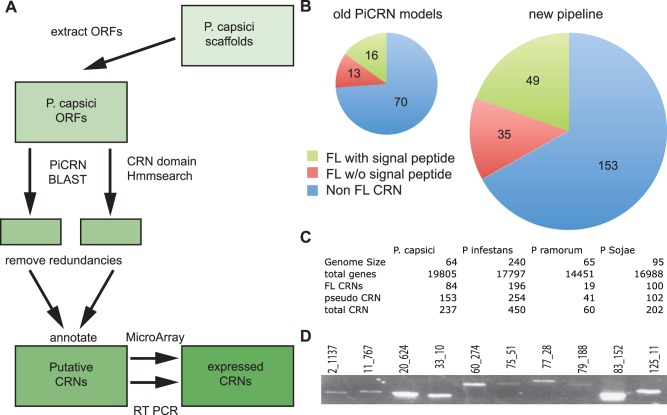
CRN annotation in *P. capsici* A) Pipeline used to re-annotate *P. capsici* CRN genes. ORFs were extracted from genome scaffolds. Known *P. infestans* CRNs and CRN HMM profiles were used to find putative CRNs. Candidates were filtered, annotated and verified via several methods. B) Visualisation of the different numbers of CRN-like genes identified using the predicted protein models of our new pipeline. Diameter of the circle represents the number of CRNs. C) Table showing gene size, gene number and number of (FL) CRNs for all sequenced *Phytophthora* spp. D) RT PCR on randomly selected CRN genes confirms presence on cDNA.

We devised a new pipeline for CRN identification and verification (Figure 1A). This pipeline was applied to raw genome sequence data and uses two separate search methods to identify putative CRN coding genes. We performed 6-frame translations of genome sequence scaffolds and extracted putative open reading frames (ORFs, >300 nt) from the *P. capsici* draft genome. BLAST searches with a set of verified *P. infestans* CRN coding genes [37] and HMMer searches employing models described previously [16] were used to identify putative CRN domains. This yielded a set of 587 putative ORFs, each encoding a CRN-like sequence. Filtering out physical redundancies and subsequent semi-manual annotation resulted in a total set of 237 CRN gene models, a significant improvement over previous predictions (Figure 1B) and resembles the numbers of CRNs found in other *Phytophthora* species (Figure 1C). To test the robustness of our pipeline, we analysed *Pythium ultimum* genome sequences and found 57 CRN-like ORFs in this species, including 16 CRN-like ORFs described during the original genome annotation. 10 other PuCRNs were not picked up by our pipeline. Closer examination revealed that these CRN-like ORFs were either too short (8) to be considered CRN gene according to our parameters or lacked a proper LFLAK motif (2). To check for false positives we also applied the pipeline on the genomes of diatoms, *Phaeodactylum tricornutum* [25] and *Thalassiosira pseudonana* [26] and plants *Arabidopsis thaliana* and *Solanum lycopersicum* (tomato) [*38*]. One gene was flagged in tomato, as it shares similarity for the kinase domains, but lacks the characteristic LXLFLAK motif in the N-terminus. In all other control species CRN genes were not identified.

**Figure 2:**
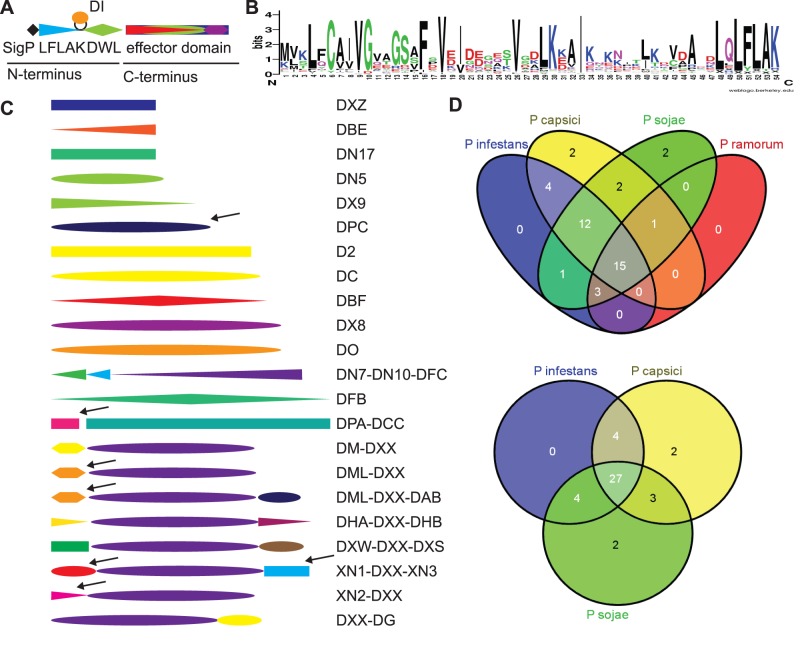
CRN domains in *P. capsici* A) Graphical representation of a CRN gene. A typical CRN gene has an N-terminus consisting of a Signal Peptide, LFLAK domain, containing the LxLFLAK-motif containing domain and DWL domain, containing the HVLVVVP-motif, in some cases a small DI domain is inserted between LFLAK and DWL domains. The C-terminus contains the effector domain and shows large variation in domain structure. B) Sequence logo representing *P. capsici* LFLAK domain. This LFLAK domain shows very close homology to the one from *P. infestans*. C) Variation of C-terminal domains found in full length CRNs of *P. capsici.* Arrows indicate domains that are unique in full length genes in *P. capsici*. D) Venn diagrams showing the distribution of CRN domains amongst different *Phytophthora* spp.

Analogous to other classes of intracellular effectors, CRNs are modular proteins that often carry a canonical secretion signal followed by a conserved N-terminal LXLFLAK motif, required for secretion and delivery of effectors into host cells [39]. In addition to the LXLFLAK motif, a highly conserved HVLVVVP motif, that defines the N-terminal DWL domain, marks a major recombination site that is followed by diverse C-termini that specify effector function (Figure 2A). We took advantage of this typical CRN architecture and defined full-length CRNs as proteins that carry the LXLFLAK motif within their first 66 N-terminal amino acids, feature the HVLVVVP motif and carry an additional effector domain. Manual inspection of all CRN-like candidates resulted in identification of 84 full-length CRN coding genes (35% of total). Prediction of signal peptides by means of SignalP [32] indicate the presence of canonical secretion signals in 58% of the predicted full length CRN proteins. It has previously been observed that CRN genes do not always contain canonical signal peptides. TMHMM searches [33] failed to identify any transmembrane domains in the full length effector set (cut-off e-value 1e-5).

To verify our predictions we performed semi-quantitative PCR on cDNA derived from RNA isolated from infected *N. benthamiana* leaf samples. We randomly selected 10 *Crn* genes and designed primers to amplify the full-length genes (Figure 1D). RT-PCR analyses yielded amplification products with the expected size in each case, suggesting that our predicted gene models were accurate. Sequencing of each amplicon confirmed amplification of the correct predicted gene and indicated the predicted sequences did not contain introns.

### The *P. capsici* Genome Encodes both Conserved and Unique Effector Domains

CRNs are modular proteins consisting of a conserved N-terminus carrying the conserved LFLAK and DWL domains. CRN C-termini on the other hand are highly diverse and are thought to specify effector functions (Figure 2A). Indeed, alignments of N-terminal regions from our full length CRN set and subsequent generation of sequence logos revealed that PcCRN N-termini share key features with those identified in other oomycete species (Figure 2B). As expected, both LXLFLAK and HVLVVVP motifs were found in the majority of N-termini and further sequence similarity was evident between *P. infestans* and *P. capsici* N-termini. These results are consistent with the observation that conserved N-terminal regions are required for secretion and delivery of CRN effectors inside host cells.

Previously, Haas *et al.* [*16*] defined a domain structure for the *Phytophthora* CRN effector repertoire, based on sequence similarity. From these studies, 36 domains were defined after semi-automated alignment and analyses of conserved protein regions with unknown functions. 33 of these domains were found exclusively in C-terminal regions. We used these CRN domain models to assess their occurrence in *P. capsici*. HMM searches and subsequent manual assignment of CRN domains to our effector set showed that 30 C-terminal effector domains were present in the full *P. capsici* CRN complement. Of those 30 effector domains, 25 were present in at least one full length CRN, showing that collectively, *Phytophthora spp.* maintain a conserved but diverse effector domain repertoire.

We further investigated *P. capsici* effector domain composition in the predicted full length PcCRNs and identified 6 novel C-terminal domains in 7 combinations (Figure 2C). One CRN protein, for which we could not assign a known domain model, was found to carry a novel domain (we have called DPC) at its C-terminus. DPC is only present in full-length *P. capsici* CRN proteins, whereas in *P. sojae* and *Py. ultimum* this domain is only found in pseudogenes. In addition to the DPC domain, we identified DPA as a new domain that co-occurs with the C-terminal DCC domain in *P. capsici*, but cannot be found in other oomycetes. Similarly we found four additional domains that exclusively form full length genes with the DXX domain (named XN1, XN2, XN3 & DML). Amongst these domains, XN2 appears unique to *P. capsici* whereas XN3 includes singleton SN4 from *P. infestans*. Figure 2D shows Venn diagrams indicating the number of domains that occur in the genomes of the sequenced *Phytophthora* spp. These analyses show that as many as 38 domains occur in the genomes of at least two species, and 27 domains occur in all three closely related species, *P. capsici, P. infestans and P. sojae.*

These results indicate that although the CRN protein family forms an ancient and conserved protein family in the oomycetes, specific effector domains have emerged, reflecting on-going effector innovation underpinning host-pathogen co-evolution and pathogenesis.

### CRN Domain Expansion may be Linked to Hemi-biotrophy and Necrotrophy

**Figure 3:**
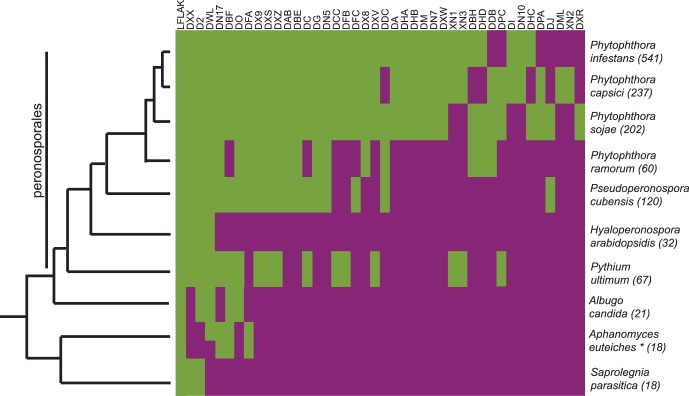
Occurrence of CRN domains in different oomycete species. Green = domain present, purple = domain absent. Total number of CRN-like genes is given between brackets. The tree left is adapted from Blair et al [44] and Thines and Kamoun [45].

We obtained evidence for this notion by super-imposing domain distributions onto a simplified phylogenetic tree describing relationships amongst sequenced oomycetes (Figure 3). Application of our HMM model set (Haas *et al.,* 2009 and this study) identified CRN domains present in publicly available genome sequences, covering a wide range of oomycete pathogens. Our searches identified previously annotated, as well as some previously un-annotated *Crn* genes. These analyses strongly suggest that the occurrence of described CRN domains follows oomycete phylogenetic relationships with only a few ancient domains shared between all species and a large group of novel domains common between *Phytophthora* species (Figure 3). To correct for a possible (evolutionary) bias caused by the use of *Phytophthora* derived CRN HMM models, we included distal LFLAK domain sequences from *P. ultimum* to remake HMM models. Subsequent searches revealed that the use of HMM model had no effect on overall *Crn* gene identification outcomes. Taken together, these results indicate that CRN domain expansion and diversification appears to have occurred after emergence of the *Peronosporales* lineage.

Interestingly, domain expansion appears to have occurred in pathogens that feature necrotrophy in their infection cycle. Domain expansion is evident in hemi-biotrophic *Phytophthora* spp but does not occur in the biotrophic pathogen *Hyaloperonospora arabidopsis*. We also see expansion in *Pythium ultimum*, which is considered a necrotrophic species, although other *Pythium* species are hemi-biotrophs [17], [40]. Thus there is evidence that CRN expansion may be correlated with an infection cycle that includes necrotrophy. Further genome sequencing from obligate biotrophs will confirm or refute this hypothesis.

### Gene Expression Analyses Defines Two Classes of CRN Effectors

To investigate expression of our predicted *Crn* gene models, we performed reverse transcriptase PCR (RT-PCR) using cDNA samples derived from a *P. capsici* infection time course on tomato (*Solanum lycopersicum*). Samples were taken from a non-infected control and at 0, 8, 16, 24, 48 and 72 hours after infection and individual time course samples were used for PCRs.

**Figure 4:**
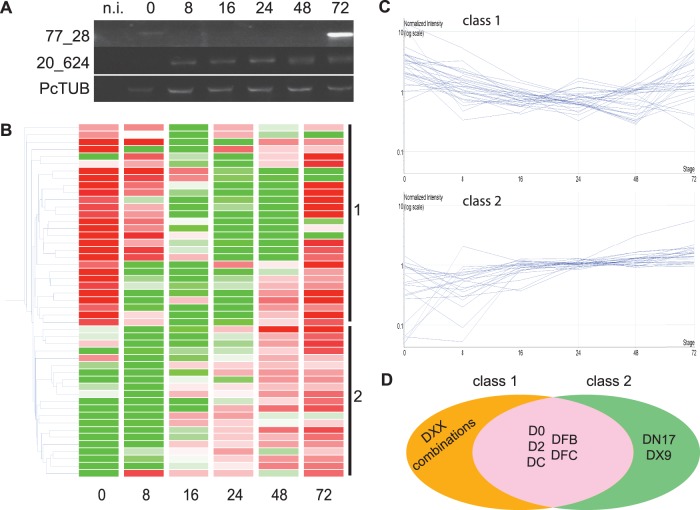
CRN gene expression upon infection A) RT-PCR on cDNA from tomato infected with *P. capsici.* Samples were collected on selected timepoints (hours post infection). B) Heat map showing expression pattern for full length *P. capsici* CRN genes. Green is down regulated, Red is upregulated compared to the median of each sample. Gene classes are indicated on the right. Numbers at the bottom indicate the time after infection that the samples were taken. C) Expression profiles for CRN genes group in two different classes. Class 1 show genes that are upregulated compared to their mean expression values directly upon inoculation and after downregulation increase in expression in the later time points. Class 2 genes have upon inoculation expression values lower than their mean, but expression goes up during the course of infection, generally in the latest stages. D) Venn diagram showing the composition of both expression classes. Genes with DXX domain (combinations) all sit in class 1.

We noted that our *Crn* coding genes show contrasting expression profiles (Figure 4A). One gene (77_28) appeared expressed at 0 hrs after infection, suggesting expression in zoospores and cysts, followed by a significant drop in subsequent biotrophic stages. Another gene however (20_624) did not show expression in the early time points but featured upregulation in the later stages of infection (Figure 4A). These results suggest the presence of distinct *Crn* gene expression patterns during *P. capsici* infection and disease progression.

To substantiate and extend these results to the full *P. capsici Crn* gene repertoire, we assessed *Crn* transcriptional changes in microarray gene expression datasets generated from a *P. capsici*-tomato infection time series (Jupe *et al*., *in preparation*). We examined the expression profiles of *Crn* genes in the array dataset and found evidence of expression for 49 of our gene models (58% of full length *Crn* genes, using unique probes) as defined by detectable signal in biological replicates for at least two stages. These results further validated our CRN identification and characterisation strategy and provided us with a robust set of genes suited to classifying gene expression patterns. We assessed CRN gene expression profiles and confirmed the presence of two classes of CRNs (Figure 4B, C). A total of 28 *Crn* genes fell into one class (1) featuring high levels of expression at the early time points, a drop during subsequent biotrophic stages and expression in the later stages (Figure 4B, C). A further 21 genes fell into another class (2), showing little or no detectable expression early in infection, but accumulation of transcripts in the late infection stages (Figure 4B and Figure 4C). We investigated if certain domains were specifically expressed in the two defined patterns. We assessed the expression of all C-terminal domains that were present more than once in the full length CRN set. This revealed that most domains were represented in both expression classes (Figure 4D). However, we found that all of the *Crn* genes encoding DXX domains were in class 1, whereas those containing DN17 and DX9 were in class 2. (Figure 4D, Table S02). These results suggest that the DXX domain in particular may have functions specific to early stages of infection.

### *P. capsici* CRN Proteins Target the Nucleus and Sub-nuclear Compartments

Previous studies established the CRN protein family as a class of nuclear effectors [13]. To assess whether PcCRN proteins we identified feature Nuclear Localisation Signals (NLS), we applied NLStradamus [30], PredictNLS [31] and NoD [22] to the full length CRN set. Our analyses resulted in a combined set of 29 *P. capsici* FL CRN sequences with at least one localisation signal (Table S03), indicating that many of the CRN genes do not carry canonical NLS. Inspection of the predicted localisation signals in CRNs revealed few similarities between NLS motifs, consistent with the observation that CRN effector domains are divergent (Table S03).

**Figure 5:**
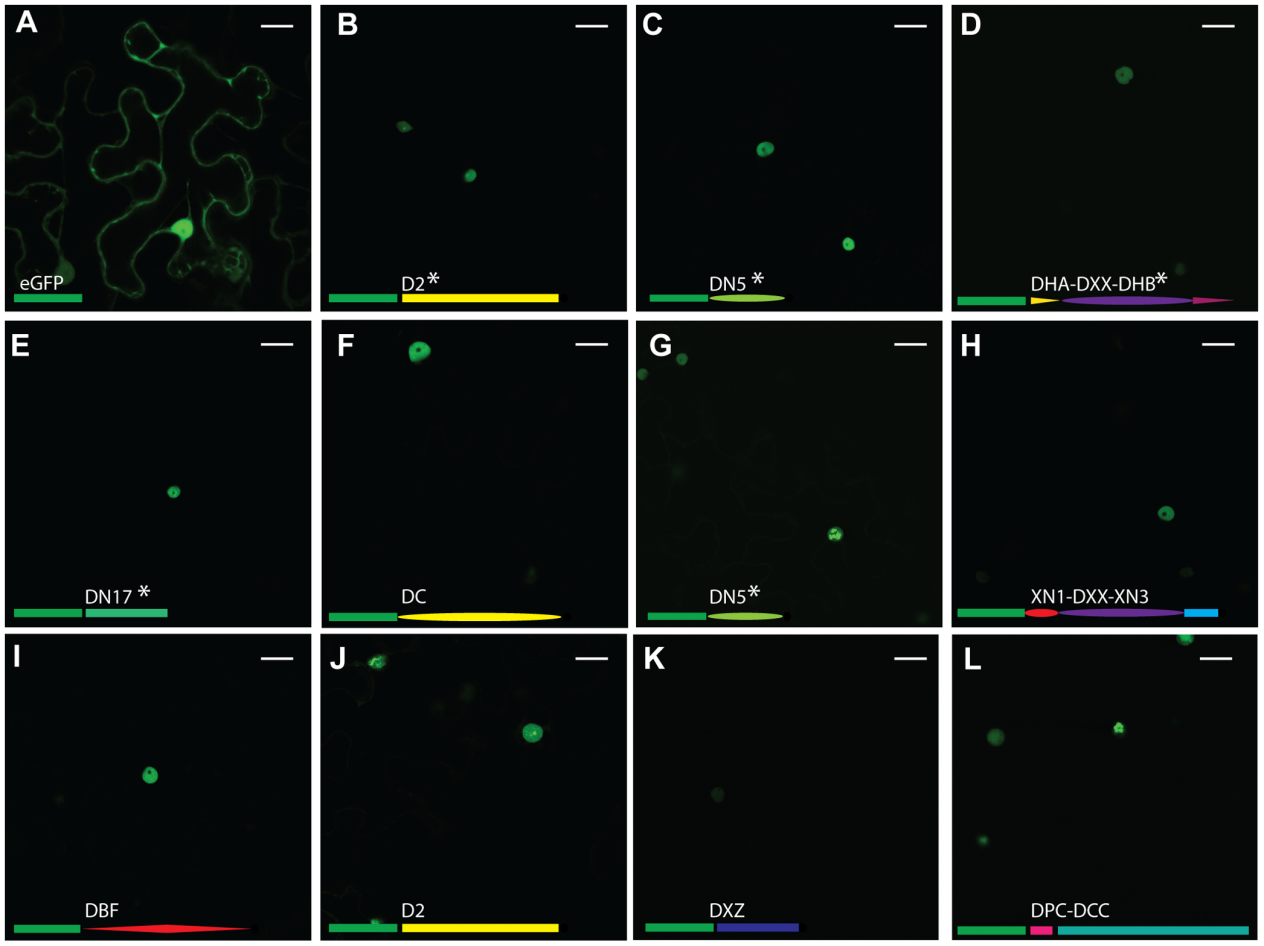
Localisation of GFP-tagged CRN C-termini. A) shows localisation of free GFP. B-L) show a diverse range of GFP-tagged CRN C-termini. B = 1_719, C = 11_767, D = 12_997, E = 20_624, F = 32_283, G = 33_10, H = 36_259, I = 60_274, J = 79_188, K = 83_152, L = 105_26. All tested CRN fusions localise to the nucleus of the cell. Different subnuclear localisations can be observed for some CRNs (B, G, J, L). The domain organisations of the C-termini are represented as fused to GFP (green rectangle) for each image. NLS are predicted to be present in the genes marked with *. Scale bar = 25 µm.

We substantiated nuclear localisation of CRN effector domains from *P. capsici in planta*. We cloned 11 PcCRN C-terminal domains and fused them to GFP. Our selection included some very divergent domains (<10% sequence similarity) and CRNs with and without a predicted NLS. For some domains, we selected multiple representatives in order to assess domain-specific localisation. *Agrobacterium tumefaciens* mediated expression of eGFP-CRN translational fusions in leaves showed specific nuclear accumulation (Figure 5). For all CRN domains tested, there was no or extremely weak GFP fluorescence in the cytosol whereas strong fluorescence emanated from plant nuclei (Figure 5). These observations contrasted with those observed in cells expressing eGFP only. Expression of eGFP consistently resulted in significant fluorescence in both the cytosol and nucleoplasm (Figure 5). Given that we sampled a comprehensive set of effectors covering the domain diversity in *P. capsici*, our results suggest that all CRNs target the host nucleus.

**Figure 6:**
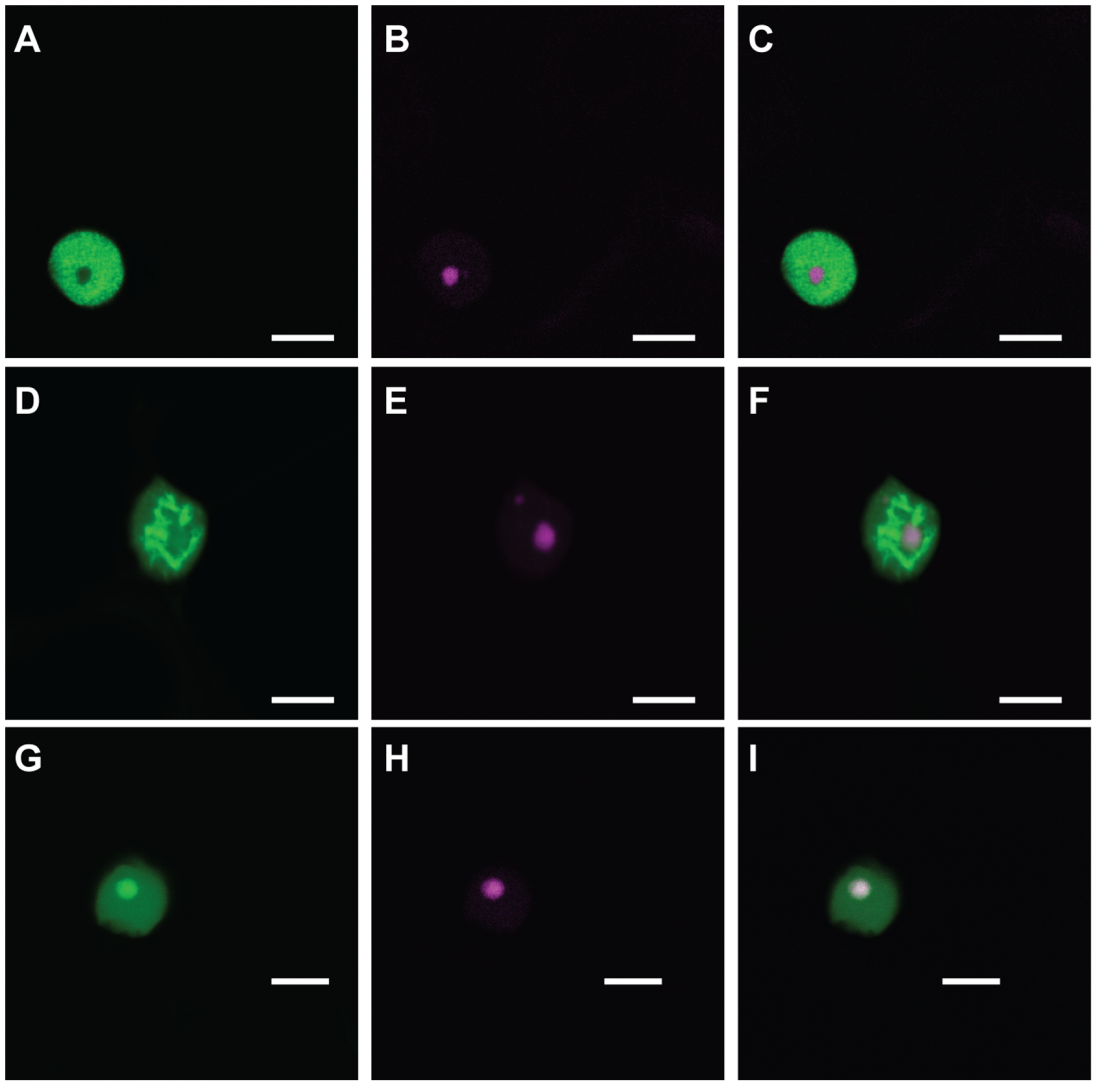
Different subnuclear localisations can be observed for CRN C-termini. First column shows GFP-tagged CRNs, second column shows RFP-tagged fibrillarin in the nucleolus and cajal body (Panel E). Third column shows overlay image. Scale bar = 10 µm.

Interestingly, we observed different subnuclear localisations for some effector domains. Whereas most domains localised to the nucleoplasm and did not enter the nucleolus, some appeared aggregate in subnuclear bodies (Figure 5 B,G,J,L). We selected two of these constructs (CRN1_719 (5B) and CRN79_188 (5J)) and CRN20_624 (5E) to represent the other CRNs and co-expressed them with RFP-tagged Fibrillarin, a marker of the plant nucleolus [36]. We confirmed that the typical CRN localisation, as seen for CRN20_624, involved CRNs entering the nucleus, but not the nucleolus (Figure 6 A–C). Two effectors containing the D2 domain, however, have different subnuclear localisations. CRN79_188 mainly localised to unknown nuclear bodies (Figure 6 D–F) and was also seen to localise around the nucleolus, whereas CRN1_719 localised in the nucleolus (Figure 6 G–I).

### Cell Death Inducing CRN Domains can be Distinguished from Virulence Enhancers

**Figure 7:**
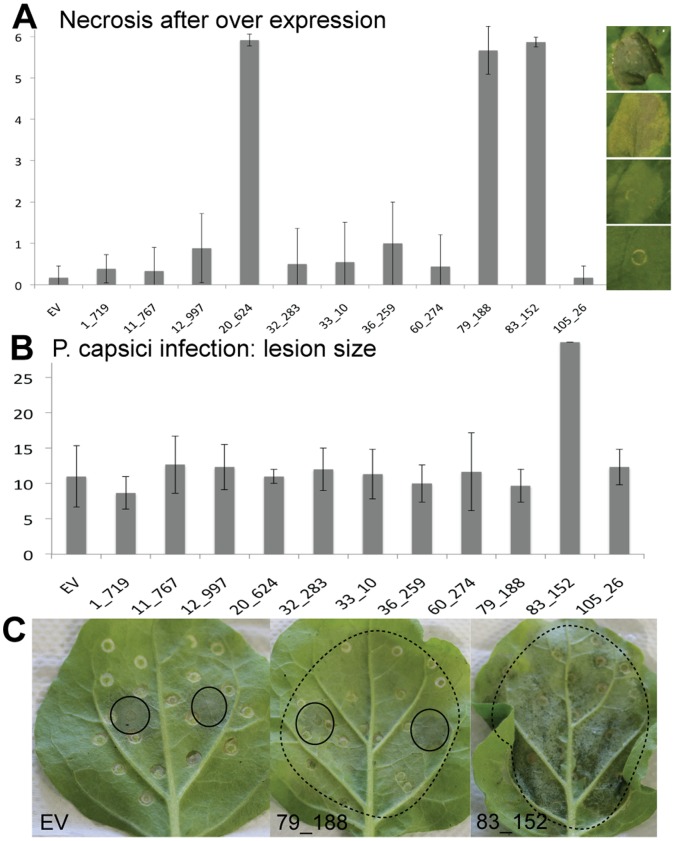
Phenotypic and functional analyses of CRN effector domains *in planta*. A) Only three CRNs caused necrosis after over-expression in plants. Bars show average values for at least three independent infiltration events with four or more infiltration sites per construct per event. B) One CRN had a direct effect on virulence of *P. capsici*. Lesion size for all other CRNs was similar to that of the empty vector (EV) control. Error bars show standard deviations within the samples. Lesion size was measured during three independent infection events using four infection sites per construct. C) Onset of necrosis was not responsible for increased virulence. Panel 2 shows necrosis onset (dotted circles) for 79_188, but no increase in *P*. *capsici* lesion size (full circles) as seen for 83_152 (Panel 3). Panel A, X-axis: necrosis score as defined by picture panels on the right, Panel B X-Axis, Lesion size in mm.

The CRNs were named after a leaf crinkling and necrosis phenotype observed upon ectopic expression in plants [12]. Recent studies however, show that this is not a universal feature of CRN proteins. Over-expression of PiCRN domains only induced necrosis in a few cases [16]. To test whether *P. capsici* CRN domains were also variable in induction of cell death, we characterized 11 CRN domains *in planta* (Figure 7). We infiltrated three leaves for each of three independent replications and scored phenotypic effects on a range from 1 (no symptoms visible) to 6 (severely necrotic, black tissue). Only 3 of the 11 domains showed a strong necrosis phenotype after four days (Figure 7A). For other domains, ectopic expression occasionally resulted in chlorosis (yellowing), but this was not consistent across replicates. Protein expression levels were established by western blot. The results were similar for each repetition. Figure S01 shows a representative blot with some variation in protein levels between constructs, however these could not be linked to phenotypic observations.

To test whether CRNs have an effect on the growth rate of *P. capsici* during infection, we performed a simple drop inoculation assay on leaves transiently expressing CRN proteins. None of the CRNs had a direct positive effect on the growth rate of *P capsici*, except for CRN83_152 (Figure 7B). Interestingly this was only one of the three CRNs that showed a necrotic phenotype. The infiltration sites for CRN83_152 were swiftly colonised by *P. capsici*, leaving wet, infected tissue, whereas leaves for CRN20_624, which also causes necrosis after several days, remained uninfected (Figure 7C).

This further indicates that the CRNs are a diverse group of effectors with a wide array of functions, some of which directly affect *P. capsici* virulence.

## Discussion

### The *P. capsici* CRN Effector Repertoire

The CRN protein family forms a class of oomycete effectors that are chronically understudied. The inability to reliably identify and classify CRN protein complements from a given pathogen has hampered functional studies in different oomycetes. Here, we applied a simple pipeline that employed 6-frame dynamic translation of raw genome sequences, followed by CRN prediction and verification studies. This approach enhanced CRN identification rates significantly for both full length genes and pseudogenes, compared to previously published results [19], thus providing a robust platform from which CRN evolution and virulence function can be further investigated. We identified 237 gene models with CRN features of which 84 were full-length. Existing descriptions of CRN domain composition and structure, allowed us to identify conserved domains, but also define novel C-terminal domains and domain configurations that may have specific roles in *P. capsici* virulence. The number of CRN coding (and pseudo) genes is similar to those found in other *Phytophthora* spp but exceed CRN repertoire size in other oomycetes such as *Saprolegnia parasitica* (18) *H. parasitica* (32) or *Py. ultimum* (67) (Figure 3). A subset of full-length genes was validated by PCR and subsequent amplicon sequencing. In addition, we obtained further support for our predictions as the majority of our predicted full length genes were found to be expressed during infection in a *P. capsici*-tomato microarray experiment (Figure 4).

### Occurrence and Evolution of CRN Domains in *P. capsici*

We assessed the *Crn* gene model complement encoded by the *P. capsici* genome. Detailed gene model annotations and comparative analyses show significant CRN domain variation and organisation within *P. capsici* and other *Phytophthora* CRN effector domains. Novel domain configurations and the presence of previously undescribed domains were found in CRN C-terminal regions. We provide evidence of dramatic expansion of the *Crn* gene family, suggesting possible roles in infection. Our analyses suggested that CRN domain innovation may be a feature of the Peronosporales lineage and pathogens that feature necrotrophy in their disease lifestyles. Further sequencing of biotrophic and necrotrophic oomycetes is required to confirm this hypothesis.

We show that the DXX and D2 domain are amongst the most widespread C-terminal CRN domains in the oomycetes. The DXX domain appears to have emerged early in oomycete evolution, yet it is part of an expanding complex. We found various effector domain configurations featuring DXX in *P. capsici*. Importantly, these included two new domains linked to DXX, suggesting that domain evolution and diversification in *P. capsici* is recent. The *P. infestans* CRN8 protein features a conserved D2 domain and has been found to carry kinase activity *in planta* which contributes to *P. infestans* virulence [23]. These results support the notion that CRN proteins play significant roles in oomycete parasitism and virulence. Future studies to identify isolate-specific domain configurations whilst assessing function in more detail will help build a better understanding of CRN evolution and function in *Phytophthora*.

### *P. capsici Crn* Genes Group in Two Distinct Expression Classes

We investigated *Crn* gene expression and observed two different expression patterns. Class 1 contains genes that are upregulated upon infection (e.g. in spores), drop down in biotrophy and show increased expression again in later stages, when sporulation is initiated. Class 2 shows no detectable expression in the very early stages, but increase after infection is established. Closer examination revealed that the DXX coding *Crn* genes exclusively exhibit class 1 expression patterns. We therefore hypothesise that the ancient DXX domain containing gene products play specific roles in establishing infection, whereas other domains support subsequent stages of the *P. capsici* life cycle. With the DN17 and DX9 domain encoding genes specifically falling into class 2, these domains may have specific roles in the late infection stages. The observation that both these domains are absent in biotrophic pathogens suggests a link to necrotrophy. Based on these observations, studies aimed at understanding pathogen lifestyles should include a detailed functional assessment of DN17 and DX9 domains.

### CRNs Localise and Target Nuclear Compartments

Previously, studies on *P. infestans* CRN proteins revealed localisation to the host nucleus, suggesting that the CRN protein family form a class of nuclear effectors [13]. CRNs used in that study contained 4 different domains and all contained a predicted NLS, or slightly modified form (single aa changes). Our work extends these results by demonstrating that all tested, highly divergent *P. capsici* CRN domains localise to the nuclear compartment, regardless the presence of predicted nuclear localisation signals. Our results contrast with studies on other effector families that collectively target various subcellular compartments. Although increasing numbers of nuclear effectors are being described [41], only the TAL class of effectors target the nucleus.

Besides the TAL effectors, pathogenic bacteria secrete a wide array of nucleo-modulins that manipulate a variety of animal host cell processes [42]. In plants, there is increasing evidence that important processes take place in the plant nucleus that affect virulence and immunity. The wide variety of defence related proteins, ranging from NB-LRR R proteins to cysteine proteases, found to localise in or re-localise to the nucleus, only strengthens this notion [43]. These observations lend support to the hypothesis that the host nucleus is a crucial compartment where plant-oomycete interaction outcomes are decided.

Detailed assessment of CRN localisation patterns revealed that in addition to nuclear accumulation, some CRN domains exhibited sub-nuclear localisation patterns. The majority of CRN domains tested localised to the nucleoplasm, whereas two D2 domain-containing effectors were found to accumulate in or around the nucleolus or in subnuclear bodies. These results suggest that the CRN protein family targets distinct sub-nuclear compartments and thus may perturb different components or processes in the host nucleus. There may be domain-specific sub-nuclear localisation patterns though more CRNs with D2 domains will have to be tested before this can be demonstrated.

Although all CRN proteins tested accumulate in the nucleus, Nuclear Localisation Signal (NLS) prediction exercises only identified NLS motifs in 26% of the PcCRN sequences tested. This indicates that either CRN proteins carry alternative NLS signals, not detectable by prediction software, or accumulate in the host nucleus by other means. The availability of large suites of CRN effector domains from a diverse group of pathogens, may allow application of computational strategies to identify novel sequence motifs that signal nuclear targeting.

### CRNs are Ancient Proteins with Diverse Roles in Pathology

Even though CRNs were named for their crinkling and necrosis phenotype upon their discovery, our assays show that only a small fraction of CRN domains induce necrosis in a short time frame. The observation that most CRNs do not show a necrosis phenotype after over-expression is consistent with previous findings [16]. Our results suggest that cell death induction is not a virulence function but rather a phenotypic manifestation reporting on effector activity.

Consistent with other studies, CRN domain classification may not distinguish between functions. We expressed two D2 domain-containing genes in leaves (CRN 1_719 and CRN 79_188) and only found evidence of cell death induction with one of them. Western blot analyses showed that both proteins were stable upon ectopic expression suggesting that domain sequence variations may specify function. These results agree with observations made by Liu *et al.* [21], who showed that two closely related PsCRN proteins have antagonistic functions when expressed in plants. The mechanisms by which small sequence variations within domains specify diverse phenotypic outcomes remain unclear. Regardless, these observations provide an ideal basis for structure-function studies in plants.

Only one CRN in our screen had a positive effect on *P. capsici* virulence. CRN83_152 enhances lesion growth rates in ectopic assays. However, this phenotype was unrelated to the cell death observed upon prolonged over expression of CRN83_152, as other cell death inducing CRNs do not have an enhancing effect on virulence. Although it remains unclear how virulence is boosted by CRN83_152 in these experiments, identification of its host targets may reveal processes that affect *P. capsici* disease progression.

Based on our results, we suggest that the CRNs have a more subtle role in plant-pathogen interactions than perhaps assumed after their discovery. Identification of CRN host targets may therefore not only help implicate the processes that are targeted in host nuclei, but could also lead to a better understanding of the infection process. The characterisation of CRN targets may help delineate other requirements for infection. This is particularly significant considering that CRN domains are found in all plant pathogenic oomycetes, appear to have expanded in hemi-biotrophs and necrotrophs, pre-date the RxLRs and are specifically regulated during infection. We suggest that studies aimed at further understanding the role of CRN effectors *in vivo* during infection, will reveal novel effector functions and host targets that underpin pathogen lifestyles.

## Supporting Information

Figure S1 **Western blot showing stable expressed CRN C-termini fused to eGFP as shown in** **Figure 5** **.** *CRN60_274 has lower steady state protein levels compared to all others and could only be detected using stronger chemiluminescence substrates.
(TIF)

Table S1 **Overview of all CRN coding ORFs identified in** ***P. capsici.***
(XLSB)

Table S2 **Microarray classification data for all** ***Crn*** **genes that had unique probes on our array.**
(XLSX)

Table S3 **Overview of NLS in CRNs, identified with different prediction software.**
(XLSX)

Table S4 **Primers used in this study.**
(XLSX)
