# Correction: Identification and Characterisation CRN Effectors in *Phytophthora capsici* Shows Modularity and Functional Diversity

**DOI:** 10.1371/annotation/90bd45cb-33a7-426f-a928-9ddc351b08cc

**Published:** 2013-04-02

**Authors:** Remco Stam, Julietta Jupe, Andrew J. M. Howden, Jenny A. Morris, Petra C. Boevink, Pete E. Hedley, Edgar Huitema

There was an error in the article title.

The correct title is: Identification and Characterisation of CRN Effectors in *Phytophthora capsici* Shows Modularity and Functional Diversity

The correct citation is: Stam R, Jupe J, Howden AJM, Morris JA, Boevink PC, et al. (2013) Identification and Characterisation of CRN Effectors in *Phytophthora capsici* Shows Modularity and Functional Diversity. PLoS ONE 8(3): e59517. doi:10.1371/journal.pone.0059517

The corresponding author is Edgar Huitema, with the email address e.huitema@dundee.ac.uk

