# Supplementary figures and images for: Identification and Characterisation CRN Effectors in Phytophthora capsici Shows Modularity and Functional Diversity
Source: PLoS One. 2013 Mar 25;8(3):e59517. doi: 10.1371/journal.pone.0059517 (PMC3607596; doi:10.1371/journal.pone.0059517)

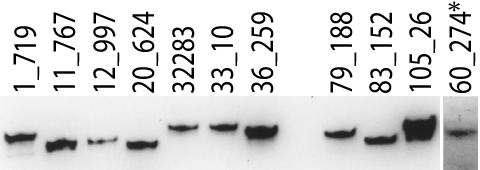

Supplement: Figure S1 — Western blot showing stable expressed CRN C-termini fused to eGFP as shown in Figure 5 . *CRN60_274 has lower steady state protein levels compared to all others and could only be detected using stronger chemiluminescence substrates. (TIF) [file pone.0059517.s001.tif]
